# Current Status of Intergroup Threats Perceived by Chinese Physicians and Its Association with Organizational Psychology, Behavior, and Well-Being during the COVID-19 Pandemic: A Cross-Sectional Survey

**DOI:** 10.3390/healthcare10101972

**Published:** 2022-10-08

**Authors:** Tao Sun, Hong-yan Yin, Shu-e Zhang, Xian-hong Huang, Bei Liu

**Affiliations:** 1 Department of Health Policy and Management, School of Public Health, Hangzhou Normal University, Hangzhou 311121, China; 2 Department of Humanities and Social Sciences, Harbin Medical University (Daqing), Daqing 163319, China; 3 Department of Health Management, School of Health Management, Harbin Medical University, Harbin 150081, China; 4 Department of Laboratorial Science and Technology &Vaccine Research Center, School of Public Health, Peking University, Beijing 100191, China

**Keywords:** Chinese physicians, intergroup threats, emotional exhaustion, psychological stress, job satisfaction, defensive medical behavior, turnover intention

## Abstract

(1) Background: Chinese physicians have encountered serious physical and verbal attacks in recent decades due to poor patient–physician relationships, leading to a broad spectrum of negative consequences. This study aims to assess the status of intergroup threats perceived by physicians and explore its association with organizational psychology, behavior, and well-being during the COVID-19 pandemic. (2) Methods: We conducted a cross-sectional online survey with physicians from November to December 2020 in three provinces: Heilongjiang Province, Henan Province, and Zhejiang Province, in China. A total of 604 physicians were recruited to complete an anonymous questionnaire. There were 423 valid questionnaires. (3) Results: We developed a 25-item intergroup threat scale with four dimensions: interest damage, performance impairment, value derogation, and unjust sentiment. Internal consistency reliability analyses showed that the four dimensions and overall scale exhibited high internal consistency (0.756–0.947). Additionally, the average scores for physicians’ perceived overall intergroup threat, interest damage, performance impairment, value derogation, and unjust sentiment were 4.35 ± 0.51, 4.24 ± 0.73, 4.33 ± 0.58, 4.22 ± 0.65, and 4.53 ± 0.55, respectively. Moreover, this study shows that the intergroup threats perceived positively by physicians were associated with psychological stress (*β* = 0.270, *p* < 0.01), emotional exhaustion (*β* = 0.351, *p* < 0.01), turnover intention *(β* = 0.268, *p* < 0.01), and defensive medical behavior (*β* = 0.224, *p* < 0.01), and were negatively associated with job satisfaction (*β* = −0.194, *p* < 0.01) and subjective well-being (*β* = −0.245, *p* < 0.01). (4) Conclusions: The newly developed scale in this study is a reliable tool for measuring intergroup threats perceived by Chinese physicians. Physicians in China were suffering high-level intergroup threats during the anti-COVID-19 pandemic, which has a significant impact on damage to organizational psychology, behavior, and well-being. Intergroup threats perceived by physicians not only enlarged the risk of emotional exhaustion and psychological stress but also threatened organizational well-being. Moreover, greater intergroup threats were associated with a lower job satisfaction, more frequent defensive medical behavior, and a higher turnover intention for physicians. The results of this study suggest that essential intervention and governance measures should be considered to protect physicians’ well-being and benefits in China, which are urgently needed.

## 1. Introduction

Recently, there has been a considerable focus on workplace violence (WPV) towards physicians worldwide [1]. WPV is a common phenomenon in Chinese hospitals [2] and its severity and frequency are acute [3]. A national survey reported that 83.4% of Chinese physicians have encountered at least one event of WPV in the hospital workplace in the preceding 12 months [4]. A recent study demonstrated that patients and their relatives caused 15.65% of physicians’ deaths in 345 hospital-based violence incidents [5]. WPV against physicians poses a range of threats leading to significant levels of harm, including physical injury, economic losses, and reputation damage [6,7]. Over time, frequent negative intergroup contact between physicians and patients will probably cause intergroup threats [7]. Thus, WPV against physicians as a typical and severe negative physician–patient contact is prone to intergroup threats. A recent report demonstrated that 64.61% of physicians reported that their legitimate rights and interests were not appropriately protected [8].

The COVID-19 pandemic has caused changes to the Chinese physician–patient relationship and strongly impacted the healthcare workforce fighting against the epidemic [9]. Chinese health professionals are widely praised by the public for volunteering at the frontline, and the frequency of violence in hospital settings has decreased significantly because they gained more respect and social approval [10,11]. Generally, while patients had positive attitudes toward physicians during the anti-COVID-19 pandemic, problems experienced at the physician–patient interaction for physicians included adding procedures to prevent infection, workload, and patients distrust in daily work. To reduce the transmission of COVID-19, hospital policies tended to align with prevention and control policies [12,13], in which patients experienced more difficulties in accessing medical care. This posed various new problems and challenges towards inactive interactions between the physician and patient [12].

Intergroup threats refer to the resources, power, values, and beliefs of one group that are challenged by the goal, development, and well-being of another [14]. Stephan and Mealy considered that intergroup threats comprise two aspects: realistic threats and symbolic threats. Realistic threats occur when the in-group’s economy, rights, physical being, safety, and wealth are threatened by the out-group; symbolic threats are regarded as intangible harm, including the loss of honor, and undermining self-identity or self-esteem of in-group members [15]. Given the experience of intergroup threats among physicians, realistic threats to physicians should include physical injury, economic losses, harm of individual development, and other tangible resources due to suffering WPV. Intangible harm, including enduring disrespect, distrust, damage to reputation, depreciation of medical service value, and others from patients or their relatives, should be considered as symbolic threats to physicians. Since the concept of intergroup threats was proposed, there have been studies on the conflicts between several groups, such as cultural majorities and minorities [16], diverse groups such as immigrants [17], and Asians and whites in the US [18]. Undoubtedly, following the emergence of WPV and stigmatization perceived by physicians, physicians frequently encounter various kinds of patient threats. However, few studies have explored intergroup threats toward physicians from patient groups, and its influence on physicians’ organizational psychology, behavior, and well-being.

Individuals can present a positive mental state after their needs are satisfied and conversely, failure to meet their needs contributes to a lower mental state [19,20]. Physicians’ needs cannot be met because they are exposed to prolonged safety and value threats, which results in a nervous state, leading to a low level of organizational psychology. A previous study showed that there was a significantly negative relationship between perceived intergroup threats and psychological well-being [21]. In threatening surroundings, individuals are also likely to exhibit poor mental health [22]. Therefore, this study assumed that intergroup threats perceived by physicians may result in negative consequences from an organizational psychology perspective, such as decreased subjective well-being, greater emotional exhaustion, and higher psychological stress.

Organizational behavior and well-being are the extensions of happiness in the organization, which reflect the individual’s overall assessment of their work [23], consisting of both emotional and cognitive aspects [24]. Organizational behavior refers to a series of behaviors in a specific organization that is prone to be shaped by the work environment, identity, and values in some complex mechanisms. A previous study argued that hazards in work environment, such as occupational and safety hazards, or sexual harassment, had a greater association with poor workplace well-being [25,26]. Several studies have demonstrated that perceived threats negatively influence organizational well-being. Ganz et al. reported that perceived threats were significantly associated with registered Israeli nurses’ unwillingness to work [27]. Another study also suggested that the feeling of stereotype threats was associated with lower job satisfaction and increased turnover intention among older employees [28]. Davis et al. found that individuals with a high level of perceived intergroup threats are more likely to respond by removing themselves from the source of potential harm [29]. Therefore, this study considers that there is a significant correlation between intergroup threats perceived by physicians and workplace well-being and behavior during the COVID-19 pandemic, such as job satisfaction, turnover intention, and defensive medical behavior.

By further extending the research scope of existing studies, this study emphasizes intergroup threats perceived by physicians and potential consequences. This study aims to examine the levels and impact of intergroup threats perceived by physicians on organizational psychology, behavior, and well-being during the COVID-19 pandemic. 

## 2. Materials and Methods

### 2.1. Subjects and Procedures

The cross-sectional survey was completed between November and December 2020. The data were collected in three steps using stratified and convenient sampling. First, the Chinese mainland was divided into three regions according to geographical location: eastern, central, and western regions of China. We used convenient sampling to select three provinces, Heilongjiang Province, Henan Province, and Zhejiang Province, from these three regions. Second, two hospitals from each province were selected, including a tertiary and a non-tertiary hospital. Third, three intermediaries between the researcher and respondent were invited as coordinators to help to recruit participants and distribute questionnaires to respondents. The intermediaries received training to understand the general integration background, purpose, meaning and significance, and respondents’ matters needing attention of this survey before questionnaire distribution. Fourth, the Chinese version of the questionnaire was created using ‘Questionnaire Star’ software. Therefore, each intermediary helped the researcher to acquire respondents by sending a website link or poster of the questionnaire to the respondents via WeChat or QQ. We adopted an anonymous method to protect respondents’ privacy. At the beginning of the questionnaire we: 1. committed to participant privacy protection, affirming that their personal information would not be divulged; 2. provided details of the study and respondents could choose to volunteer for this study. Everitt recommended that the sample size in factor analysis be at least ten times greater than the item [30]. Therefore, the minimum sample size was calculated as 350 participants in this study. According to Eng J’s method, the minimum sample size was calculated as 378 participants [31]. Considering a drop-out rate of 30% (approximately 113), the sample size of this study should be expanded to at least 491 participants. A total of 604 respondents completed the questionnaire. The inclusion criteria were: (1) clinicians working at the selected hospitals, (2) voluntary participation, and (3) informed consent from the participants. The exclusion criteria were: (1) more than three missing items, (2) recommended exclusion that was self-reported by the participants, and (3) if the participants’ completion time was less than three minutes. A total of 423 valid questionnaires were obtained with an effective response rate of 70.03%. 

### 2.2. Measurement of Intergroup Threats Perceived by Physicians 

We used the intergroup threat scale developed for this study to measure intergroup threats perceived by physicians. The scale included 25 items divided into four dimensions: benefit impairment, performance impairment, value derogation, and unjust sentiment. Each item was rated on a 5-point Likert scale (1 = strongly disagree and 5 = strongly agree), with a higher score representing a higher level of perceived threats. Cronbach’s α coefficient of the intergroup threats in this study was 0.947, which indicates good construct validity.

The intergroup threat scale was developed according to scale development theory and applications by Devellis in 1991 [32]. First, we conceptualized the intergroup threats perceived by physicians by combining social phenomena in the Chinese context, semi-structured interviews, and literature reviews regarding intergroup threats [14]. Second, following semi-structured and expert consultations, we generated the corresponding items for measurement. Thirty-five initial items were created from the above strategies. We carefully considered wording modifications throughout the process. The items were rated on a 5-point Likert scale (1 = strongly disagree, 5 = strongly agree). Third, we used survey and statistical analysis to examine the validation of the scale.

### 2.3. Measurement of the State of Physicians’ Organizational Psychology

We assessed organizational psychology using measures of subjective happiness, psychological stress, and emotional exhaustion by the participants. Organizational psychology is a complex and multifactorial construct [33]. A single indicator was a poor measure of organizational psychology. Therefore, three indicators, subjective happiness, stress, and emotional exhaustion, were selected to represent physicians’ whole state of organizational psychology, and those indicators have been used to measure organizational psychology in the Chinese context [34]. Considering the physician’s workload, subjective happiness and psychological stress were separately measured by a single item. We measured emotional exhaustion using a subscale of the Burnout Inventory Scale. The following single-item measure of subjective happiness was used: “In general, do you currently feel happy?” The response was coded on a 5-point Likert scale (1 = “very unhappy” to 5 = “very happy”), with a higher score representing better well-being. This subscale has been extensively used in the Chinese context [34]. Psychological stress was assessed by the following question: “Stress means a situation in which a person feels tense, restless, nervous, or anxious or is unable to sleep at night because their mind is troubled all the time. Do you currently feel this kind of stress?” Responses were recorded on a 5-point Likert scale, ranging from (1 = not at all to 5 = very much). This single-item measure of psychological stress has good validity and sensitivity [35]. The emotional exhaustion questionnaire used a subscale of the Burnout Inventory Scale, originally compiled by Maslach et al. [36]. This subscale was revised to the Chinese version by Li et al. as an important component of the burnout inventory [37] and has been widely used on the Chinese mainland with qualified validity and reliability [38]. Five items were coded from (0 = never) to (6 = frequently), with higher scores indicating a higher level of emotional exhaustion. Cronbach’s α coefficient of the scale in this study was 0.958.

### 2.4. Measurement of Physicians’ Organizational Behavior and Well-Being

Considering that organizational behavior and well-being are multi-dimensional [28,39], it is difficult to measure these using a single dimension. Therefore, three items in monitoring “job satisfaction,” “defensive medicine behavior,” and “turnover intention” were selected to represent the physicians’ organizational behavior and well-being. These indicators were measured using a single-item question, which had been used in a previous study in the Chinese context [34]. The following single-item measure of participants’ job satisfaction was used, “Overall, how satisfied are you with your current job?”, which was rated on a 5-point Likert scale, (1 = strongly dissatisfied and 5 = strongly satisfied), with higher scores indicating higher job satisfaction. The validity and reliability of this scale have been proven in previous research [40]. Defensive medicine behavior was measured with one item, “In the past year, have you conducted any defensive medical actions while providing medical care service to patients, such as referral, multiple tests, or avoidance of high-risk patients?” We used a rating scale (1 = never to 5 = frequently). Higher scores indicated that physicians practiced more defensive medical services. The question “In the past year, have you had any ideas of leaving your current position?” was used to measure the turnover intention of physicians. The responses were rated on 5-point Likert scale (1 = never and 5 = frequently), where a higher score indicated a higher level of turnover intention. 

### 2.5. Statistical Analysis

We used a descriptive statistical analysis to assess the variables of demographic characteristics and status of the intergroup threats perceived by physicians. We adopted a principal component analysis to analyze dimensionality reduction and construct the dimension of the intergroup threat scale. We conducted internal consistency tests to examine the reliability of the scale. Pearson’s correlation coefficient analysis was used to evaluate the correlations between intergroup threats perceived by physicians, organizational psychology, behavior, and well-being. All factors influencing intergroup threats perceived by physicians in the univariate analysis (*p* < 0.05) were entered into the hierarchical regression analysis. We used SPSS (version 26.0, IBM, Armonk, NY, USA) to analyze the results. Moreover, *p* < 0.05 (two-tailed) indicated statistical significance. 

## 3. Results

### 3.1. Demographic Information of Participants

The demographic characteristics of the respondents are presented in Table 1. In total, there were 423 participants, including 224 (52.96%) females and 199 (47.04%) males, 170 (40.19%) Master’s degree and 190 (44.92%) attending physicians. Among the participants, 152 (35.93%) were between the ages of 31 and 35, 189 (44.68%) received a monthly income between CNY 5001 and CNY 9000, 177 (41.84%) had between four and 10 years of service, and 214 (50.59%) worked daily for between 8 and 10 h. Moreover, most respondents (78.72%) were married or cohabiting, and most (80.85%) were from tertiary hospitals. We used analysis of variance (ANOVA) to measure the differences in demographic variables. The results show that there were no significant differences.

### 3.2. Exploratory Factor Analysis and Reliability Analysis of the Construct of Intergroup Threats Perceived by Physicians

We conducted an exploratory factor analysis to explore the structure of intergroup threats. The results show that the Bartlett spherical test coefficient was 0.000, representing a level of significance. The KMO value was 0.953 (>0.9). These results indicate that the factor analysis is considered appropriate, illustrating several common factors among the 35 items.

We conducted a principal component analysis with varimax rotation to assess the intergroup threat scale’s construct validity. A total of 25 items from 35 were included in the formal scale. Some items with low factor loading or cross-loading were deleted in the multi-step process of exploratory factor analysis. The principal component analysis suggested a 4-factor structure, accounting for 65.83% of all variances. Moreover, the highest factor loading was 0.848 and the lowest 0.467. The details of the structure and factor loading of each item are listed in Table 2. The results indicate that the questionnaire had a good construct validity.

We used Cronbach’s α coefficient to evaluate the reliability of the intergroup threat scale. The results indicate that the overall Cronbach’s α coefficient of intergroup threat was 0.947. Cronbach’s α coefficients of the dimensions interest damage, performance impairment, value derogation, and unjust sentiment were 0.756, 0.797, 0.910, and 0.941, respectively. The results indicate that the questionnaire had a good reliability, as shown in Table 3. 

### 3.3. The Average Scores of Different Types of Intergroup Threats Perceived by Physicians 

The average overall and four domain scores for intergroup threats are reflected in Table 4. The average overall intergroup threat score was 4.35 ± 0.51, in the range of 2.60–5.00. The average score of unjust sentiment was the highest at 4.53 ± 0.55. Moreover, the average scores of interest damage, performance impairment, and value derogation were 4.24 ± 0.73, 4.33 ± 0.58, 4.22 ± 0.65, respectively. These results demonstrate that physicians suffer a high level of intergroup threats.

### 3.4. Relationships among Study Variables

Table 5 displays the means, standard deviations, and Pearson correlation coefficients of the variables. The results show that intergroup threats positively correlated with emotional exhaustion (*r* = 0.347, *p* < 0.01), psychological stress (*r* = 0.269, *p* < 0.01), and defensive medical behavior (*r* = 0.225, *p* < 0.01), and negatively correlated with subjective well-being (*r =* −0.249, *p* < 0.01), job satisfaction (*r* = −0.198, *p* < 0.01), and turnover intention (*r* = −0.515, *p* < 0.01).

### 3.5. Hierarchical Linear Regression Analysis

We conducted a hierarchical linear regression analysis to test the effect of intergroup threats on organizational psychology, behavior, and well-being. The results are presented in Table 6 and Table 7. To eliminate the effects of demographic variables such as gender, marital status, age, service years, education, title, and monthly income, they were regarded as control variables (see Table 6). The results show that intergroup threats were negatively associated with subjective well-being (*β* = −0.245, *p* < 0.01, *M*_2_), and positively associated with emotional exhaustion (*β* = 0.351, *p* < 0.01, *M*_4_) and psychological stress (*β* = 0.270, *p* < 0.01, *M*_6_). These findings indicate that the higher intergroup threats perceived by physicians were associated with a low level of organizational psychology during the COVID-19 pandemic.

Table 7 shows that intergroup threats are negatively associated with job satisfaction (*β* = −0.194, *p* < 0.01, *M*_8_) and positively associated with defensive medical behavior (*β* = 0.224, *p* < 0.01, *M*_10_) and turnover intention (*β* = 0.268, *p* < 0.01, *M*_12_). This indicates that intergroup threats are negatively associated with physicians’ organizational behavior and well-being during the COVID-19 pandemic. 

## 4. Discussion

### 4.1. Chinese Physicians’ Exposure to High-Level Perception of Intergroup Threats

The results show that the average score of overall intergroup threats perceived by physicians was 4.35 ± 0.51 (>3). This indicated that physicians perceived high-level intergroup threats from patients and their families during the COVID-19 pandemic. The mean scores of the four types of intergroup threats perceived by physicians, including interest damage, performance impairment, value derogation, and unjust sentiment, were 4.24 ± 0.73, 4.33 ± 0.58, 4.22 ± 0.65, and 4.53 ± 0.55, respectively. The scale measuring intergroup threats was a new tool; therefore, it was difficult to compare the physicians level of perception of intergroup threats and that of other groups. Generally, some studies have shown similar findings. For example, Zhang et al. conducted a survey on the prevalence of workplace violence towards nurses using a seven-item scale and found that 75.4% of Chinese nurses reported that they had exposure to violence [41]. According to the intergroup threat theory, the existing greater intergroup threats perceived by physicians is understandable. Given the high incidence of WPV towards physicians [4], interpersonal interactions between physicians and patients in China are tense [42]. Both physicians and patients are dissatisfied with the healthcare system [43]. A previous Chinese survey reported that > 90.0% of Chinese physicians had experienced at least one event of WPV during the preceding 12 months [44]. In other words, many physicians have negative interactions with patients or their relatives, contributing to the damage of tangible or intangible resources, resulting in physicians’ high perception of intergroup threats. Additionally, Chinese physicians are exposed to various negative commentaries from news reports and public opinion on social media [45,46], thus indirectly destroying the physician–patient relationship, and increasing conflict and confrontation. During the COVID-19 pandemic, physicians were exposed to many infected individuals, increasing their workload [47]. Hence, the current results recommend that policymakers raise immediate concerns regarding physicians facing intergroup threats in China.

### 4.2. The Negative Association between Intergroup Threats and Physicians’ Organizational Psychology 

The results show that the perception of intergroup threats was associated with emotional exhaustion, psychological stress, and subjective well-being, which is consistent with the previous study [21]. This suggests that intergroup threats perceived by physicians are associated with organizational psychology. The physicians’ perceptions of intergroup threats have great explanatory power in predicting emotional exhaustion. According to the conservation of resources theory, individuals are prone to making significant efforts to acquire, invest, and protect valued resources [48]. On the contrary, once emotional exhaustion emerges, resources tend to be threatened or lost [48]. Physicians experienced a loss of resources, facing threats such as damaged tangible resources, physical resources, occupational stigma, and unacknowledged professional value that aggravated their emotional exhaustion, characterized by a sense of tiredness, fatigue, and a lack of energy [49,50]. The conservation of resources theory also posits that threatened and depleted resources are a crucial determinant of psychological stress [51], indicating that the perception of threats should be regarded as stressors [21], thereby fostering greater psychological stress. Furthermore, intergroup threats may spillover into physicians’ lives. This is more likely to decrease the overall appraisal of their lives, followed by lower subjective well-being.

### 4.3. The Negative Association between Intergroup Threats and Physicians’ Organizational Behavior and Well-Being

The results show that intergroup threats perceived by physicians negatively associated with workplace well-being. Intergroup threats perceived by physicians negatively associated with job satisfaction and positively associated with defensive medical behavior and turnover intention, which was consistent with findings in other studies. Research has shown that perceived professional reputation damage is positively associated with frequent defensive medical behaviors and intense turnover intention [34]. Another study also proved that stereotype threats positively associated with low job satisfaction and high turnover intention [28]. In China, physicians devote a great amount of energy and effort to meet patient needs. However, most patients respond with disrespect, unfriendly interactions, and various threatening signals, causing a sense of effort–reward imbalance and low attachment, and resulting in physicians probably displaying defensive medical behavior to avoid lawsuits [52]. Previous studies also reported similar findings, suggesting that intergroup threats are positively associated with avoidance and protective behaviors [53,54]. The existing literature indicates that individuals tend to adjust their behavioral responses in the face of threats using avoidance, and offensive and defense behaviors [15]. These behaviors consist of increased examination of patients’ conditions, apprehension to accept patients with serious conditions, and increased referral of patients to other hospitals [55]. To escape an unfriendly work environment, victims are likely to leave the healthcare industry and join other industries, which is a major concern [56,57]. According to the UN Sustainable Development Goals (SDGs) and the World Health Organization Health Workforce 2030 strategy, the density of physicians is 27.2 per 10,000 population in China compared with central Europe, eastern Europe, and central Asia’s average of 38.3 [58]. Simultaneously, some scholars have found that most Chinese medical students, as future physicians, had a very strong dropout intention [59]. To protect physicians, interventions and governance measures should be implemented, including setting entry or exit screening, installing an alarm button for physicians, launching a zero-tolerance policy, and introducing law to protect personal safety for health professionals, to reduce workplace violence in hospital settings [60]. Additionally, government-provided free medical care for patients infected with COVID-19 in mobile cabin hospitals relieved the physician–patient relationship tension, which will enlighten future health reforms [61]. Correspondingly, we recommend that Chinese hospital managers and policymakers take positive actions to protect the interests and well-being of both physicians and patients through seasonable coping with the intergroup threat crisis. For example, the government should introduce concrete policy measures to control health expenditure and increase financial investment in the public hospital to reduce physician–patient conflicts on economic benefits, thus increasing physician–patient trust, which is more likely to decrease occurrences of intergroup threats. 

## 5. Limitations

This study contributed to a new viewpoint to explore the physician–patient relationship with positive theoretical and practical significance. However, this study has some limitations. First, we collected data using an online survey and self-report method, which is likely to produce response bias due to negative effects or social desirability. Second, the non-random sampling method potentially causes sample bias, which can affect the study results. Third is the cross-sectional nature of the variables. Fourth, the scale for measuring intergroup threats, perceived by Chinese physicians, was developed. However, a prospective study of the tool was missing in this study. Fifth, although the sample population characteristics, such as sex and title, were similar to the national physician demographic data from the “China Health Statistical Yearbook 2020”, edited by the National Health Commission of China [62], there were some differences in age and service years between the two, which can influence the overall results. Furthermore, many single-item-measured variables were used to evaluate the association with variables, which may influence the validity and reliability. Although a novel tool for the intergroup threat scale was developed and preliminarily validated, further research is needed to test its validity and reliability in different cultural contexts. Moreover, although we studied the physician–patient relationship from a new perspective, the impact of COVID-19 was not reflected in the study design due to its sudden outbreak. Therefore, the effect of COVID-19 pandemic on our results is not perfectly predicted. 

## 6. Conclusions 

The newly developed scale, in this study, is recommended when measuring intergroup threats perceived by Chinese physicians, and those associated with organizational psychology, behavior, and well-being during the COVID-19 pandemic. Physicians who perceived a high degree of intergroup threat reported not only more serious emotional exhaustion and psychological stress, but also less organizational well-being. Moreover, intergroup threats were associated with decreased job satisfaction, and increased defensive medical behavior and physicians’ intention to leave the job. We recommend that essential intervention and governance measures should be considered to urgently protect physicians’ well-being and benefits in China.

## Figures and Tables

**Table 1:** Demographic characteristics of the respondents (*n* = 423).

Characteristics	*n*	%
Gender		
Male	199	47.04
Female	224	52.96
Marital status		
Married/Cohabitation	333	78.72
Unmarried/Single	81	19.15
Divorced/Widowed/Other	9	2.13
Age (years)		
≤30	86	20.33
31–35	152	35.93
36–40	94	22.22
41–45	55	13.00
46–50	25	5.91
≥51	11	2.60
Education		
College degree or below	6	1.42
Bachelor’s degree	119	28.13
Master’s degree	170	40.19
Doctoral degree	128	30.26
Title		
Without professional title	25	5.91
Resident physician	100	23.64
Attending physician	190	44.92
Associate chief physician	82	19.39
Chief physician	26	6.15
Monthly income (CNY)		
≤5000	83	19.62
5001–9000	189	44.68
9001–15,000	97	22.93
15,001–20,000	38	8.98
>20,000	16	3.78
Years of Service		
≤3	73	17.26
4–10	177	41.84
11–15	84	19.86
16–20	46	10.87
21–30	37	8.75
≥31	6	1.42
Daily working hours		
<8	130	30.73
8–10	214	50.59
>10	79	18.68
Hospital level		
Tertiary hospital	342	80.85
Non-tertiary hospital	81	19.15

**Table 2:** Rotated component matrix of all items.

Items	Factor Loading
Factor 1: Interest Damage	
In physician–patient conflicts, physicians are prone to face the stress of financial compensation	0.848
Patients and their family members are prone to claim extra financial benefits from the physicians through practices out of various “ulterior motives” (e.g., making trouble, “YINAO”)	0.786
In physicians–patient conflicts, patients and their family members are prone to threaten the physicians’ personal safety	0.606
Factor 2: Performance Impairment	
In the work, physicians’ professional suggestions are prone to be questioned or even repudiated by patients and their family members	0.467
Patient and their family members are prone to propose various unreasonable requests in the process of treatment, leading to an increase in physicians’ unnecessary workload	0.715
Patients and their families deliberately make difficulties for physicians, which will increase the difficulty of physicians’ work	0.762
Patients and their families are prone to misrepresent the situation of disease, making it harder for physicians to make a diagnosis	0.761
Exposure to physician–patient disputes is prone to hinder physicians’ career development, such as interrupted professional title and career promotion	0.489
Factor 3: Value Derogation	
Patients and their family members believe that the hospital provides services under the incentive only derived by economic interest, thereby contributing to the derogation of physicians’ occupational values	0.645
Patients and their family members are prone to spreading malicious message about physicians, such as taking kickbacks and red envelopes, and poor morality	0.719
To date, the career image of physicians in the mind of patients and their family members is becoming worse than before	0.757
In the process of physician–patient disputes, patients and family members are prone to choosing to smear physicians’ reputation by using not physician-friendly information in social media	0.708
Patients and their family members are prone to distrusting physicians’ technical and professional skills during diagnosis and treatment	0.716
Patients and their family members are prone to distrusting physicians’ professional ethics or the code of conduct	0.701
Patients and their family members are prone to exhibit less empathy to doctors by ignoring physicians’ survival needs and respectable needs	0.533
Patients and their family members are prone to show some disrespect behaviors towards physicians during diagnosis and treatment	0.623
Factor 4: Unjust Sentiment	
Media reports about workplace violence towards physicians contribute to increased risk of threats to physician’s safety through the imitation effect of violence phenomenon	0.592
In the news and public opinion, the standpoints and suggestions tend to appeal to the patients’ rights and interests rather than those of physicians	0.770
Patients are prone to being seen as a vulnerable group in the news and public opinion, which contributes to unfair preference for physicians	0.812
Medical service capacity and technology are prone to being exaggerated by media reports, leading to increased physicians’ stress from the comment of public opinion in medical practice	0.765
In the news and public opinion, people are prone to forcing physicians’ behavior choices by proposing moral coercion, then overlooking physicians’ essential needs	0.819
In Chinese media, there are a lot of false news for slandering physicians, including untruthful statements, accusations or irrational complaints	0.778
Media coverage tends to report unfavorable images about physicians rather than positive images, resulting in the worsening of the public reputation of physicians	0.683
The unfavorable media coverage about physicians destroys patients’ trust in physicians’ professional ethics and code of conduct	0.783
The media coverage tends to report physicians’ profit-seeking behaviors even if it is a minority phenomenon, but seldom or never discuss physicians’ professional value	0.774

**Table 3:** Coefficients of internal consistency.

Dimensions	Cronbach’s α
Overall intergroup threat	0.947
Interest damage	0.756
Performance impairment	0.797
Value derogation	0.910
Unjust sentiment	0.941

**Table 4:** Mean and standard deviation of intergroup threat (*n* = 423).

Threat styles	Mean ± SD	Range Min–Max
Overall intergroup threat	4.35 ± 0.51	2.60–5.00
Interest damage	4.24 ± 0.73	1.00–5.00
Performance impairment	4.33 ± 0.58	2.00–5.00
Value derogation	4.22 ± 0.65	2.13–5.00
Unjust sentiment	4.53 ± 0.55	2.56–5.00

Note: SD = Standard Deviation; Min = Minimum; Max = Maximum.

**Table 5:** Correlations among study variables (*n* = 423).

Variables	Means	SD	1	2	3	4	5	6	7
Intergroup threat	4.35	0.51	1						
Subjective well-being	3.05	0.75	−0.249 **	1					
Emotional exhaustion	3.05	1.41	0.347 **	0.544 **	1				
Psychological stress	3.15	0.89	0.269 **	0.498 **	0.584 **	1			
Job satisfaction	2.94	0.82	−0.198 **	0.668 **	−0.474 **	−0.430 **	1		
Defensive medical behavior	2.13	0.92	0.225 **	0.227 **	0.303 **	0.276 **	0.205 **	1	
Turnover intention	2.42	1.07	−0.515 **	0.515 **	0.560 **	0.515 **	0.534 **	0.309 **	1

Note: ** *p* < 0.01; SD = Standard Deviation; Correlation is significant at the 0.01 level (2-tailed).

**Table 6:** Hierarchical linear regression models for organizational psychology (*n* = 423).

Variables	Subjective Well-Being		Emotional Exhaustion		Psychological Stress	
	*M*_1_(*β*)	*M*_2_(*β*)	*M*_3_(*β*)	*M*_4_(*β*)	*M*_5_(*β*)	*M*_6_(*β*)
Control variables						
Gender	−0.033	−0.016	−0.011	−0.036	−0.070	−0.089
Marital status	0.032	0.027	−0.094	−0.087	−0.081	−0.076
Age	−0.125	−0.132	−0.082	−0.072	−0.037	−0.030
Service years	0.046	0.073	0.053	0.015	0.062	0.032
Education	−0.153 **	−0.159 **	0.093	0.102	0.101	0.108
Title	0.020	0.029	0.084	0.071	0.067	0.057
Monthly income	0.184 *	0.169 **	−0.034	−0.012	−0.107	−0.090
Independent variable						
Intergroup threat		−0.245 **		0.351 **		0.270 **
F	2.605 *	5.802 **	1.725	9.099 **	2.255 *	6.291 **
R^2^	0.026 *	0.083 **	0.012	0.133 **	0.020 *	0.091 **
ΔR^2^	0.042 *	0.059 **	0.028	0.121 **	0.037 *	0.072 **

Note: ** *p* < 0.01; * *p* < 0.05; F = equality of variances; *β* = standardized coefficients; R^2^ = the fit of the model; ΔR^2^ = R^2^-changed; *M*_1_, *M*_3_, *M*_5_: the influence of demographic variables on the Subjective happiness, Emotional exhaustion, and Psychological stress; *M*_2_, *M*_4_, *M*_6_: the influence of Intergroup threat on the Subjective happiness, Emotional exhaustion, and Psychological stress.

**Table 7:** Hierarchical linear regression models for organizational behavior and well-being (*n* = 423).

Variables	Job Satisfaction		Defensive Medical Behavior		Turnover Intention	
	*M*_7_(*β*)	*M*_8_(*β*)	*M*_9_(*β*)	*M*_10_(*β*)	*M*_11_(*β*)	*M*_12_(*β*)
Control variables						
Gender	−0.046	−0.032	−0.095	−0.111 *	0.104 *	0.085 *
Marital status	0.021	0.017	−0.053	−0.049	−0.119 *	−0.114 *
Age	−0.311 **	−0.317 **	−0.005	0.001	−0.114	−0.107
Service years	0.182	0.204	0.096	0.072	0.104	0.074
Education	−0.187 **	−0.192 **	0.014	0.019	0.083	0.090
Title	0.069	0.075	0.073	0.065	0.085	0.075
Monthly income	0.210 **	0.198 **	0.029	0.043	−0.093	−0.077
Independent variable						
Intergroup threat		−0.194 **		0.224 **		0.268 **
F	4.676 **	6.393 **	3.158 **	5.730 **	2.362 *	6.313 **
R^2^	0.057 **	0.093 **	0.035 **	0.082 **	0.022 *	0.092 **
ΔR^2^	0.073 **	0.037 **	0.051 **	0.049 **	0.038 *	0.070 **

Notes: ** *p* < 0.01; * *p* < 0.05; F = equality of variances; *β* = standardized coefficients; R^2^ = the fit of the model; △R^2^ = R^2^-changed; *M*_7_, *M*_9_, *M*_11_: the influence of demographic variables on the Job satisfaction, Defensive medical behavior, and Turnover intention; *M*_8_, *M*_10_, *M*_12_: the influence of Intergroup threat on the Job satisfaction, Defensive medical behavior, and Turnover intention.

## Data Availability

Extra data are available by email: hydsuntao@126.com.
